# Mixture toxicity, cumulative risk, and environmental justice in United States federal policy, 1980–2016: Why, with much known, was little done?

**DOI:** 10.1186/s12940-021-00764-5

**Published:** 2021-09-17

**Authors:** Robert Hunt Sprinkle, Devon C. Payne-Sturges

**Affiliations:** 1 School of Public Policy, University of Maryland, College Park, MD 20742 USA; 2 Maryland Institute for Applied Environmental Health, School of Public Health, University of Maryland, College Park, MD 20742 USA

**Keywords:** Mixture toxicity, Cumulative risk, Environmental justice, Environmental Protection Agency, National Institute of Environmental Health Sciences, Pesticides, Herbicides

## Abstract

Toxic chemicals — “toxicants” — have been studied and regulated as single entities, and, carcinogens aside, almost all toxicants, single or mixed and however altered, have been thought harmless in very low doses or very weak concentrations. Yet much work in recent decades has shown that toxicants can injure wildlife, laboratory animals, and humans following exposures previously expected to be harmless. Additional work has shown that toxicants can act not only individually and cumulatively but also collectively and even synergistically and that they affect disadvantaged communities inordinately — and therefore, as argued by reformers, unjustly. As late as December 2016, the last full month before the inauguration of a president promising to rescind major environmental regulations, the United States federal environmental-health establishment, as led by the Environmental Protection Agency (EPA), had not developed coherent strategies to mitigate such risks, to alert the public to their plausibility, or to advise leadership in government and industry about their implications. To understand why, we examined archival materials, reviewed online databases, read internal industry communications, and interviewed experts. We confirmed that external constraints, statutory and judicial, had been in place prior to EPA’s earliest interest in mixture toxicity, but we found no overt effort, certainly no successful effort, to loosen those constraints. We also found internal constraints: concerns that fully committing to the study of complex mixtures involving numerous toxicants would lead to methodological drift within the toxicological community and that trying to act on insights from such study could lead only to regulatory futility. Interaction of these constraints, external and internal, shielded the EPA by circumscribing its responsibilities and by impeding movement toward paradigmatic adjustment, but it also perpetuated scientifically dubious policies, such as those limiting the evaluation of commercial chemical formulations, including pesticide formulations, to only those ingredients said by their manufacturers to be active. In this context, regulators’ disregard of synergism contrasted irreconcilably with biocide manufacturers’ understanding that synergism enhanced lethality and patentability. In the end, an effective national response to mixture toxicity, cumulative risk, and environmental injustice did not emerge. In parallel, though, the National Institute of Environmental Health Sciences, which was less constrained, pursued with scientific investigation what the EPA had not pursued with regulatory action.

## Background

Toxic chemicals — “toxicants” — in environmental media act and interact and are acted upon, some persisting unchanged, others changing predictably or unpredictably, uncounted numbers mixing in uncontrolled proportions with numerous traces co-accumulating in animals, including humans, with low-dose individual and synergistic effects reported by reputable laboratories. These facts and their implications have been studied for decades [1], in recent years with some urgency, questions now probing gestational, developmental, and latent reproductive and oncogenic consequences. Concern has grown especially pronounced within, and on behalf of, disadvantaged communities with presumptively chronic toxicant exposures and demonstrably poor health [2]. Citizens there have been advancing inequity arguments on an environmental-justice basis and implicating chemical and nonchemical stressors on a cumulative-risk basis [3].

Mixture toxicity, cumulative risk, and related findings — low-dose effects, synergism, endocrine disruption, inordinate exposures in poorer-paying jobs and poorer neighborhoods — have elicited various responses: disinterest, dismissal, confusion, alarm. What these findings have not elicited has been a consistent response within the United States federal environmental-health establishment. Within that establishment are leading and subsidiary units. The Environmental Protection Agency (EPA) monitors environmental quality, enforces standards in accordance with statutes and rulings, regulates pesticides, maintains inventories of chemicals and toxic releases, and conducts laboratory testing, scientific research, and environmental education. The National Institute of Environmental Health Sciences (NIEHS) conducts and funds research probing the human health effects of environmental exposures; its Superfund Research Program (SRP) studies contaminated sites and their remediation, and its National Toxicology Program (NTP) develops analytical innovations and maintains a registry of carcinogens. The Centers for Disease Control and Prevention (CDC) includes two environmentally oriented subsidiaries: the Agency for Toxic Substances and Disease Registry (ATSDR), an advisory and non-regulatory unit focused on hazardous waste, and the National Center for Environmental Health (NCEH), whose core commitment is to protect from certain environmental hazards the public’s vulnerable subpopulations: the young, the old, and the disabled [4] but not disadvantaged communities as such. Between and among these units responsibilities have overlapped, and contrasts have emerged.

As late as December 2016, the EPA, bearing primary federal environmental regulatory responsibility, had not developed a coherent strategy to mitigate mixture toxicity and cumulative risk, to alert the public to their plausibility, or to advise leadership in government and industry about their ramifications. Then, a month later, with the inauguration of a new president, environmental regulation itself became the first focus of an intensive effort to loosen restrictions on industrial and commercial activities, an effort, in sum, to accept more risks and reduce fewer [5]. We have examined the 36 years — six presidential administrations, from Carter through Obama — prior to this discontinuity.

During this period, 1980–2016, when initiatives might have advanced, stasis prevailed, even as an empirical premise for action had been converging, as sampled here, from three different directions: field study, epidemiological study, and experimental study.

Field study had found abnormal development, including intersex anatomy, physiology, and behavior, among aquatic animals and their predators in watersheds subject to point-source [6] and non-point-source [7, 8] pollution. Study of surface-water toxicants followed by experimental study of fish exposed embryonically to those toxicants in environmentally documented concentrations had found the adverse effects of a mixed exposure unpredicted by toxicant additivity [9–13].

Epidemiological study had found differential risk for urogenital abnormalities in children of countries similar in most respects but hosting dissimilar dominant industries [14, 15], and mothers in these countries had been found to express breast milk showing country-specific signatures of multiple endocrine disrupting chemicals [16]. Cohort comparison had found reproductive abnormalities, notably oligospermia, in men known to have been exposed to dioxins during gestation in Seveso, Italy [17], or to perfluorinated alkyl acids during gestation in Denmark [18]. Surveillance in the United States had found these particular toxicants and various other toxicants co-occurring — somatically “mixing” — in most pregnant women sampled. Blood drawn from women, pregnant and nonpregnant, during the National Health and Nutritional Examination Survey (NHANES) 2003–2004 had been assayed for 163 environmental toxicants of 12 classes in combinations limited to 71 toxicants at a time; pregnant women assayed for 71 toxicants carried a burden ranging from 35 to 60 with a median of 50 [19].

Experimental study had often found disturbing effects. Mixtures of homologous oil shale components had proved additively toxic to marine bacteria, but mixtures of non-homologous components, even of structural isomers, had proved synergistically toxic [20]. Many environmental toxicants had exhibited low-dose non-threshold effects [21–24]. Systematic review had found numerous examples of environmental toxicants producing hormone-like nonmonotonic dose–response curves [25] and low-dose effects [26]. These observations had made little sense in toxicology, but they had made good sense in endocrinology [27] and promised to do so in allied fields, provoking an aggressive rebuttal by private consultants funded by the American Chemistry Council [28, 29]. In rat pups following developmental exposures to mixtures of mechanistically dissimilar antiandrogens in doses individually expected to show no adverse effect, anatomical abnormalities had been found, implying synergistic, rather than simply additive, harm [30]. Exposure to mixed environmental toxicants in rats had correlated with reproductive pathologies manifesting across three generations in environmentally unexposed pups, with exposure-specific epigenetic biomarkers linking ancestral exposures to adult-onset disease in subsequent generations [31]. Similar work likewise had found that “a mixture of plastic derived compounds … can promote epigenetic transgenerational inheritance of adult onset disease” [32]. Different in focus and scale had been the Halifax Project, a global carcinogenicity review by 173 co-authors. Its conclusion, reported in 2015, had been admonitory: “Our current understanding of the biology of cancer suggests that the cumulative effects of (non-carcinogenic) chemicals acting on different pathways that are relevant to cancer, and on a variety of cancer-relevant systems, organs, tissues and cells could conspire to produce carcinogenic synergies that will be overlooked using current risk assessment methods” [33].

Why, with much known, was little done? This question, to our knowledge, has not previously been posed across disciplines and decades. Its answer should help explain how scientific understanding and regulatory response diverged and how their divergence may yet be narrowed.

## Methods

We accessed holdings of the National Archives and Records Administration to examine documents, published and unpublished, deposited by or relating to the EPA and allied bodies from 1980 to 1986, the period when mixtures policy was forming and mixtures guidelines were being written. We analyzed the development, provisions, and consequences of six federal statutes enacted from 1972 through 2016, one Supreme Court ruling from 1980, and one presidential Executive Order from 1994. To study the legislative history of the Food Quality Protection Act, the federal statute that in 1996 introduced cumulative-risk and aggregate-risk obligations, we accessed United States Senate archives; complementary House of Representatives archives remained under embargo. We inspected one herbicide manufacturer’s internal communications released fortuitously through court order. We reviewed policy statements and framework documents and seven National Research Council advisory studies and assessed their influence. We searched PubMed.gov for “environmental mixture toxicity”, “cumulative risk”, “environmental justice”, and numerous narrower associated terms, concentrating on the 1980–2016 period and examining scientific evidence, topical reviews, and expert commentaries that either motivated or illuminated the policy history being probed. We did not conduct a systematic review of these literatures, nor did we conduct a meta-analysis. To supplement objective findings, and in a manner compliant with requirements of our Institutional Review Board, we interviewed four topic experts, three of them participants in the development and implementation of the policies investigated. The authors throughout collaborated to identify and pursue emergent themes.

## Findings

We present our findings narratively over time, tracing statutory, judicial, methodological, political, scientific, and regulatory factors as they came to prominence and as they interacted. Interviews are placed where most pertinent.

### Complexities encountered

Scientific interest in mixture toxicity had increased enough by 1952 to warrant comprehensive review and theoretical specification, with interactions — synergism and antagonism — modeled mathematically and cumulative risk promoted presciently as a topic for future study [34]. Yet policy interest was slow to grow. What “slow” indicated might have been skepticism, but it might also have been disorientation. Thousands of industrial chemicals, many unstudied either toxicologically or epidemiologically, were mixing and reacting in uncertain numbers and at unknown concentrations in varying physical conditions while subject to microbial and higher-order metabolism. Then, altered or not, mixed or not, they were being contacted in exposures once expected to be harmless but now suspected to be harmful, whether during gestation and childhood or later over the lifespan. And all this had been happening, gradually or repeatedly, on watershed and airshed scales, even on whole-population scales, making epidemiological inference overly dependent on historical trends. Most disorienting might have been the growing realization that the ability to metabolize these chemicals safely was not uniform; it varied substantially, even drastically, species-to-species and person-to-person [35].

The EPA, formed 2 December 1970, focused initially on pollution at its sources: stack emissions, industrial and municipal effluents, pesticides applied to crops, and chemicals spilled onto land or into water. Immediate problems were not subtle, and “risk” was rarely mentioned. As multiple low-dose exposures to unfamiliar and poorly characterized chemicals became appreciated as commonplace, a new concept, “risk assessment”, took shape [36]. EPA’s core analytical discipline became toxicology, the laboratory science committed to characterizing noxious exposures and their effects in living organisms. A noxious chemical pollutant was to be managed either by banning it outright or by making its further use tolerable through regulation, often directing that best available technologies be employed for production, storage, application, and disposal. This second approach, “command and control” with continued use, conceded accumulation but, if vigorously applied, could slow it down. Carcinogens got the most attention, but, public concern aside, industry expected a scientific rationale for any regulation imposing economic costs [37].

The EPA soon had additional responsibilities and, regarding chemical formulations, peculiar constraints. The Federal Insecticide Act of 1910 had been amended as the Federal Insecticide, Fungicide, and Rodenticide Act of 1947 (FIFRA) [38], essentially a truth-in-labeling law [39]. FIFRA was then to be amended transformationally by the Federal Environmental Pesticide Control Act of 1972 [40], which moved FIFRA’s administration from the Department of Agriculture to the EPA and adjusted the law’s focus to match the agency’s protective mission.

In the new FIFRA, foundational definitions appeared. “Active ingredient … in the case of a pesticide” was defined as “an ingredient which will prevent, destroy, repel, or mitigate any pest”. “Inert ingredient” was defined as “an ingredient which is not active”. And “pesticide” itself was defined as “(1) any substance or mixture of substances intended for preventing, destroying, repelling, or mitigating any pest, and (2) any substance or mixture of substances intended for use as a plant regulator, defoliant, or desiccant”. A pesticide, then, even if a mixture of ingredients “active” and “inert”, was, for regulatory purposes, unitary, and in this respect the EPA’s purview was to be “any pesticide if it contains any new active ingredient or if it would entail a changed use pattern” [40].

Then, in 1976, the Toxic Substances Control Act (TSCA; colloquially, “Tosca”) [41], entered law and named the EPA as its administrative agency. While seeming to regulate “chemical substances and mixtures” in detail, TSCA, in Sect. 3, excluded from the definition of “chemical substance” not only “any mixture” — an intuitive exclusion — but also “any pesticide (as defined in the Federal Insecticide, Fungicide, and Rodenticide Act) when manufactured, processed, or distributed in commerce for use as a pesticide”. FIFRA had said a pesticide could be a chemical substance or a mixture of chemical substances, but now in TSCA a pesticide would not be considered a chemical substance [42]. Pesticides, then, could neither be mixtures nor form mixtures.

TSCA went on, still in Sect. 3, to define “mixture” tortuously and, one may suspect, tendentiously:

(8) The term “mixture” means any combination of two or more chemical substances if the combination does not occur in nature and is not, in whole or in part, the result of a chemical reaction; except that such term [“mixture”] does include any combination which occurs, in whole or in part, as a result of a chemical reaction if none of the chemical substances comprising the combination is a new chemical substance and if the combination could have been manufactured for commercial purposes without a chemical reaction at the time the chemical substances comprising the combination were combined.

(9) The term “new chemical substance” means any chemical substance which is not included in the chemical substance list compiled and published under Sect. 8 (b) [43].

Untwisting these passages suggests their authors’ intent. No mixture could “occur in nature”, so at least one of any mixture’s chemical components had to be an industrial substance. No component could be “the result of a chemical reaction” without meeting two requirements. The “result” could not be “a new chemical substance”, a term paragraph (9), above, would define as a chemical not officially listed, and “the combination” had to be one that “could have been manufactured for commercial purposes without a chemical reaction”. So, really, all components had to be industrial substances, and that conclusion may tell why no guidance explained how two or more industrial substances could be declared a mixture when found among myriad natural substances; no guidance was needed. An environmental mixture could not be, in TSCA terms, a “mixture” if its components included chemical substances altered in the environment. Nor could the still toxic breakdown products of two different industrial substances constitute a mixture.

These paragraphs, shrewdly crafted, could not have made environmental sense to drafters and legislators and regulators but would have made sense of other sorts. Polluters’ liability risks would have been easier to foresee, and to avoid, with mixtures defined restrictively. And the EPA’s responsibility to study real mixtures in real environments would have been diminished.

Might statutory intent still be honored if mixtures were studied in laboratories? Yes. TSCA expressly required the testing of a mixture per se when prior knowledge of its individual components seemed a poor guide to understanding: “If the Administrator finds that … in the case of mixtures, the effects … on health or the environment may not be reasonably and more efficiently determined or predicted by testing the chemical substances which comprise the mixture[,] the Administrator shall by rule require that testing be conducted” [44]. To the administrator’s many authorities, explicit and implicit, had been added one more explicitly, but, given the narrowness with which “mixture” was being defined, this particular authority hardly mattered. In practice, the EPA was being authorized, absent the administrator’s objection, to assess any mixture as if its separately identifiable components were acting in isolation both from nature and from each other, and those components — with “new chemical substances” excluded — would already be known. Were the administrator to object or were in-house agency practice to move toward more speculative evaluation, insight could still be gained, but constraints on regulation would remain.

On 2 July 1980, for reasons unrelated to mixtures, risk assessment began assuming a more prominent role in federal regulatory process. The Supreme Court ruled in “the Benzene Decision” — *Industrial Union Department, AFL-CIO v. American Petroleum Institute* — that toxicant-specific quantitative risk assessment was necessary to justify regulatory intervention at any particular exposure level [45]. The Occupational Safety and Health Administration (OSHA), a unit in the Department of Labor, had tried to restrict occupational exposure to benzene to “the lowest technologically feasible level that will not impair the viability of the industries regulated”. A 5-to-4 court majority instead required OSHA to prove that exposure at a specific level would pose a significant risk before prohibiting exposure at-or-above that level. No campaign to minimize emissions or exposures would henceforth be permitted, nor would any insistence that pollution-control methods be improved simply because better technology had become available. The Court, keen to unburden industry, did not have its pick of molecular protagonists, yet benzene was tragically miscast as a typical poison. As the Court was made aware, benzene was carcinogenic, so OSHA could cite no threshold level below which exposures would be safe. Nevertheless, as three of the five justices in the majority complained, “The burden was on OSHA to show … a significant risk. Here, OSHA did not even attempt to carry such burden of proof” [45]. Any agency motivated to formulate a lowest-technologically-feasible-level standard and explain its rationale cogently would be welcome to try, with seven of nine justices predisposed to be persuadable.

In the mean time, which would extend to the present day, the ruling would remain in force. The lowest-technologically-feasible-level standard would be disallowed for any specified toxicant, at least when alone and, presumably, when mixed. Demonstrating harm at-or-above any certain exposure would not imply that all lower exposures would be harmless. Yet all lower exposures would have to be allowed.

The Benzene Decision was pivotal. Quantitative risk assessment became necessary prior to regulatory intervention, which could be justified only when based on a study detecting a risk unambiguously. A study powered to detect a risk justifying intervention at a lower exposure would presumably have to be larger and slower and more costly and, if a trend toward toxicity were to fail tests of statistical significance, would prove useless. The Benzene Decision would make coming concerns — toxicity at very low doses, complexity of environmental mixtures, endocrine disruption in fetal life, and so forth — difficult to address in regulation, even difficult to envision in regulatory context.

Five months after the Benzene Decision, on 11 December 1980, President Jimmy Carter signed into law the Comprehensive Environmental Response, Compensation, and Liability Act of 1980 (CERCLA), better known as “Superfund”. CERCLA organized the EPA’s approach to reclamation following industrial or accidental contamination. It tasked the EPA with assessing and managing severely polluted sites by removing hazardous materials and remediating their residual effects. Since many of these sites had been contaminated by multiple toxicants over long periods, remediation would require that chronic exposure to low-dose environmental chemical mixtures be understood in toxicological terms.

Also coming in 1980, although not in print until 30 April 1981, was a National Research Council (NRC) case study of United States Coast Guard officers’ exposures to cargo-hold vapors and the toxicological interactions made likely by such exposures [46]. Herein toxicological interaction was defined, mathematical models were specified, and data gaps were delineated. Persistent under-attention to toxicants’ interactions was noted, ruefully [47], but “[u]ntil more specific information becomes available, it seems that the most productive course to follow to determine limits for multiple exposures is to assume additivity and follow the guidelines for mixtures as recommended by the American Conference of Governmental Industrial Hygienists (1977)” [48]. An extensive program of laboratory research was envisioned to test toxicant pairs over their dosage ranges with explicit attention to “reactive intermediates and tests for myoneural conduction, altered behavior, two-stage carcinogenesis, mutagenesis, teratogenesis, [and] effects on reproduction” [49]. The Coast Guard was advised to “collaborate with organizations such as the National Library of Medicine, the Oak Ridge Toxicology Information Response Center, and other groups” [49]. The EPA was not mentioned, nor had it been represented among participants, but it did have this report on file [50].

Around the same time, with the NRC’s assistance, a prior disaster — a calamitous multigenerational natural experiment — was being re-examined. Appearing 17 June 1982 was the result, *Assessment of Multichemical Contamination: Proceedings of an International Workshop, Milan, Italy, April 28–30, 1981*. The disaster under review had been a 1976 industrial dioxin accident in Seveso, Italy. Among the workshop’s findings was this: “Chemicals released into the air, surface waters, and soil will generally react with other chemicals in those media. The resulting products will frequently react with other chemicals, and complex series of reaction may continue along extended physical transport pathways before the materials find semipermanent storage sites in terrestrial soils or aquatic sediments” [51]. In these two sentences was summarized the rationale that had led to Superfund.

In a different section was another sentence that should have made an abiding impression. It concerned isomers, spontaneously twinned molecules identical in atomic composition but not identical in atomic conformation — or, often, in toxicity. Many chemicals were “chiral”, meaning “handed”. They were “left-handed” and “right-handed” isomers mixed together. Manufacturers, including pharmaceutical firms, were often unaware of or unconcerned about isomerization or unable to evaluate or avoid it profitably. “This is significant for environmental chemistry”, warned the Seveso report, “as there are many cases in which different isomers of a basic structure can have vastly different biological properties, including toxicity” [52]. The EPA did have this Seveso report on file [53].

Lessons being learned in the Coast Guard and Seveso studies were ones the EPA’s toxicologists could read but not ones the EPA as an agency could apply to its regulatory mission, against whose constraints Superfund was already straining.

To help it understand its evolving responsibilities, the EPA held a workshop 29–30 September 1982 in Cincinnati, Ohio, the second day addressing “Health Assessment of Exposures to Chemical Mixtures” [54]. Only “about a half dozen Superfund-designated sites” had so far been investigated, and in “some easily identified sites” only one or two “marker chemicals” had “dominated the situation” [55], but more difficult cases had to be anticipated. Presenters favored methods ranging from site-by-site, exposure-by-exposure, subject-by-subject analysis to complex mathematical modeling of toxicant interactions. One participant objected that “Any argument for toxic interaction stands on weak ground unless compounds are naturally reactive in some manner” [56], but another objected from a contrasting perspective, saying “I was disappointed that the second day of the workshop was devoted almost entirely to 2-chemical interactions, since we live in an N-chemical world” [57]. In the second day’s summation, “It was pointed out that experimental designs for [multichemical interactions] were extremely complex, required very large numbers of [experimental] animals, and were not likely to be done. … The mathematical modeling … is quite complicated” [58]. The prevailing metaphor at this meeting was still laboratory toxicology, not yet environmental chemistry and never epidemiology or public health. No consensus emerged.

The next year, 1983, the NRC published *Risk Assessment in the Federal Government: Managing the Process* [59], a report intended for all risk-assessing agencies. Within the EPA, it was one of several unrelated reports called “The Red Book”. This particular “Red Book” was a 191-page manual, still used, that standardized risk-assessment practices. Mixture-related apprehensions remained subliminal except on two pages:

[T]he latent period between exposure and disease is long, and exposures are mixed and multiple. Thus, epidemiologic [*sic*] data require careful interpretation. Even if these problems are solved satisfactorily, the preponderance of chemicals in the environment has not been studied with epidemiologic methods, and we would not wish to release newly produced substances only to discover years later that they were powerful carcinogenic agents. These limitations require reliance on less direct evidence that a health hazard exists [60]. … The importance of exposures to a mixture of carcinogens is another factor that needs to be considered in assign [*sic*] human exposures. For example, exposure to cigarette smoke and asbestos gives an incidence of cancer that is much greater than anticipated from carcinogenicity data on each substance individually. Because data detecting such synergistic effects are often unavailable, they are often ignored or accounted for by the use of various safety factors [such as dividing a no-observed-adverse-effect-level dose, a NOAEL dose, by 10] [61].

Parenthetically, cigarette smoke, mentioned above, was not an individual substance but was already known to be a mixture of toxicants [62], even a mixture of carcinogens, including polonium-210 [63]. That irony aside, the Red Book’s emphasis continued to be assessment of the human health risks presented by exposure to single chemicals, and the EPA would adopt and maintain that emphasis.

Within a year, though, mixture-related apprehensions would cease to be subliminal. Whatever statutes required regulatory science could fulfill. Whatever statutes and the Supreme Court forbade regulatory science could avoid. But regulatory science was *science*: it needed to know. And at this juncture it knew that linear regulatory policy was about to meet non-linear unregulated nature — polluted nature — and would have to adapt.

### Guidelines proposed

Thousands of hazardous waste sites, known generically as “Superfund sites”, had been contaminated with multiple chemicals and required risk prioritization to account for mixtures. Chemical-by-chemical risk assessment, environmental toxicology’s standard deliverable, had to be rethought [64]. In January 1984, the EPA formed the Agency Working Group on Risk Assessment Guidelines, and by August a first draft of a guidelines document was finished.

Few toxicants had ever been evaluated in pairs, fewer still, if any, in complex mixtures. Would addition — adding the doses of sufficiently similar chemicals or somehow adding their effects — be good enough? Not generally; a simplifying restriction to similars would make addition practically useless in many environmental field-work settings. And as evident pharmacologically, small differences between similar molecules could generate markedly dissimilar effects; for instance, folic acid, taken as a vitamin, and methotrexate, taken as an anti-cancer drug, were almost identical — so similar that enzymes requiring the former were competitively inhibited by the latter [65]. Mathematical complexity discouraged the addition of a third chemical and precluded the addition of an *nth*; any combination assessed for cumulative risk would be arbitrarily small. Most glaringly when the subject was mixtures, addition’s limitations led the Agency Working Group to ignore interaction, whether potentiation or synergism or antagonism. In the end, the Agency Working Group chose to dismiss interaction not as a possibility but as a topic:

Previous drafts of the document contained a lengthy discussion of the biological and chemical bases of toxicant interactions. The Agency Working Group felt that the discussion detracted from, rather than added to[,] the clarity of the document, and this section was deleted. The literature cited in the document provides a detailed discussion of this issue for the interested reader [66].

The Agency Working Group conceded that a two-component “mixture of concern”, if well enough characterized, might be considered a single compound, as if it were an ordinary toxicant or carcinogen evaluated for subchronic or chronic effects; dose-additive equations could be modified and interaction could be built in rather than modeled [67]. Not recalled here was TSCA’s stipulation that the term “chemical substance” could not include “any mixture” [68]. The Agency Working Group authors went on to concede that “the default position”, which had become dose additivity, was “based on the assumption that the components in the mixture have the same mode of action and elicit the same effects”, but “[t]his [similarity] assumption will not hold true in most cases, at least for complex mixtures of systemic toxicants” [69].

With the interactions discussion deleted, with simple assumptions modeling complex realities, and with no answer for most cases, the document went out for external peer review. Misgivings were many and criticisms sharp.

Most problematic for the Agency Working Group were the comments of Ellen Silbergeld, PhD, an environmental engineer and, at the time, Chief Toxics Scientist at the Environmental Defense Fund. “You will note that these comments are not supportive”, she wrote, 23 August 1984, and then in pen added a marginal comment: “I consider them unrealistic, since their explicit limitations make them difficult to apply in most situations facing the EPA”. What she had read was a plan not to grasp an exponential reality but to patch a linear algorithm.

She continued:

I am deeply concerned by the implications of these guidelines, and I would recommend that they be withdrawn for further refinement. In their present state they are unclear and unscientific for the reasons described herein. … Clearly, many interactions of humans and the ecosystem are with mixtures, rather than pure compounds. The difficulty of dealing with this problem is compounded by the fact that we remain alarmingly ignorant of the toxicity of the majority of chemicals used in our environment … . Thus it is perhaps premature to expect that we can accurately assess the threat of mixtures of chemicals, when we partially understand only about 10% of existing chemicals taken one at a time. … Even when specific exposures can be defined — such as lead in air — these exposures occur in the context of concurrent exposures, from other sources, to a multiplicity of chemicals. Thus these guidelines are arguably the most important developed by EPA. … Unfortunately, the present guidelines fall short of providing scientifically reasonable or truly protective approaches[.] It is clearly stated … that synergism (interactions other than additive) will not be considered in these guidelines. There is little justification for this policy decision, to reject any incorporation of models of other than additive interactions among toxicants. … [I]f interactions can be demonstrated, then equations other than those built around additivity must (rather than may) be used. The further discussion of possible (undemonstrated) interferences yielding less than additivity is suggested [disingenuously] to discount this obligation [to model synergism]. This is consistent with the whole thrust of this document[, which] is to exclude quantification other than additivity. … [This is] unacceptable[.] … In my opinion, most toxicant interactions occur because two or more chemicals affect the same organ system, through action at different receptor sites or cellular targets. … [This document is] scientifically unsubstantiated … [and based upon a] pathetically incomplete database … [and is] severely limited … [by too] many unhelpful comments. … It is commendable that the document admits that the assumptions underlying dose additivity “will not hold true in most cases”. However, no guidance is given to deal with these (majority) of cases. … [This is] a remarkably inflexible statement … [I am v]ery critical of the mathematical models … [which are based on] a simplistic assumption that because two compounds may antagonize each other at one site of action, they have no other affects [*sic*] which require assessment [70].

Though Dr. Silbergeld’s analysis stood out as the stiffest challenge to the Agency Working Group’s process and product, no reviewer was wholly supportive. That said, Dr. Earl C. Spurrier, writing 11 March 1985 for the National Agricultural Chemical Association, whose member firms sold chemical formulations — pesticides — expressly intended for environmental use, did seem appreciative. He conceded that pesticides were sure to mix in unaccountable ways ineluctably associated with the food chain. But his focus was not on the role of pesticides in mixtures; his focus was on the likely future status of pesticides *as* mixtures — as mixtures *themselves* — if statutory protections were ever weakened. Dr. Spurrier saw a safe harbor just beyond the EPA’s methodological mist. Reasoning backwards from dose additivity to the similarity assumption that under special conditions might make dose additivity defensible, he asserted that pesticide formulations could not be evaluated as mixtures because their constituent chemicals, each added for a reason, were all *dissimilar*, one from another [71]. Dr. Spurrier’s paradox, however preposterous, was fairly stated. It elicited no rebuttal. Implicit was this hypothesis: even if statutes were amended to acknowledge pesticide formulations as mixtures, the EPA would not be able to adjust its methods. It would still regulate pesticides as single entities accompanied by nonentities, “declared active principal ingredients” sharing containers with supposedly “inert” co-formulants.

### Guidelines approved

The following month, on 22–23 April 1985, the EPA’s Science Advisory Board Risk Assessment Guidelines Review Group convened in Washington as the Complex Mixtures Panel.

Fourteen panelists, five from the EPA, three from industry, attended the first day [72], which began acrimoniously. The EPA could present few relevant data, not even exposure data, and was pressing for chemical-mixtures risk-assessment guidelines based only on studies of single agents [73]. Dr. Robert Scala, a biologist representing the Exxon Corporation, quickly remarked that hydrocarbon mixtures, with as many as “240 identifiable materials”, exemplified additivity’s inadequacy [74]. The most salient comments on the guideline’s first draft, he added, had been Dr. Silbergeld’s. “They were beautifully written by a woman scientist who clearly understands what she’s talking about” [75].

Assessment of Superfund sites typically presented mixtures involving many chemicals, but panelists had been “required to do something”, said Dr. Jerry Stara of the EPA, even while studies of two or three chemicals and “the whole interaction base for reactivity together — in other words, synergism” were still underway [76]. Dr. Scala found additional targets, objecting “That a number which has its uncertainty plus or minus the universe isn’t a particularly valuable number [77]… I mean, it’s the worst of all possible worlds, where you have written your regulation and now you’re going to go down to the laboratory and do some experiments to justify it. … I mean, is the public pressure, is the societal need so great that we cannot take a few years on complex mixtures and do some experiments and erect the theory? … Get some real-world materials, go out and test them, and see what you learn. I’m sorry — that’s what science is about” [78]. Dr. Stara agreed, noting that Love Canal — the Hooker Chemical Company dump site near Niagara Falls, New York, notorious as Superfund’s first reclamation project — mixed 26 chemicals, which was far more than guidelines could ever model [79]. Dr. Scala then objected that Love Canal’s dangers had been “blown out of all proportion. … Essentially there was nothing proved, particularly in the reproductive area. Nothing proved. Nothing established scientifically [80]. … Science has been prostituted to come up with some kind of a figure which was really essentially a policy judgment” [81]. Dr. Stara conceded that “we are pushed so hard — and maybe I shouldn’t say this much for the record — by the regulators to produce something” [82].

Discussion turned to Dr. Silbergeld’s objections, which were accepted as valid but around which some way had to be found to avoid abandoning the guidelines project entirely. Dr. Stara had spoken with Dr. Silbergeld and reported that if “a dozen changes” were to be made “then she would agree to putting the guidelines out, you know. … Actually, we don’t need to worry about that right now because she will be out of commission for a year or two”. The meeting transcript here recorded “(Laughter)”, after which another panelist from the EPA added, “Probably three months or so” [83]. Dr. Silbergeld was in week 29 of a normal term pregnancy [84].

Five panelists attended the second day [85], which began well. Dr. Silbergeld had now “sort of bought in”, Dr. Scala reported [86]. She might support the guidelines document if it appended a “technical report” [87].

Four panelists, Dr. Stara the one excluded, proceeded to confer and write [88]. Dr. Scala, who by now was designing the group’s default assessment algorithm, explained, “When I default to no risk assessment, it says: You have data on only a small fraction of components” [89]. Dr. James Whittenberger, an occupational-health expert and the panel’s chairman, responded “They’re worried about acceptance of that one in the agency, although they say they agree with it” [90]. Dr. Scala then reassured Dr. Whittenberger that EPA professionals knew they were unprepared to perform risk assessment on complex mixtures but cautioned that their “employers” and “agency policy” might nonetheless prevail [91]. Dr. Scala offered an insight: “The other guidelines — mutagenicity, carcinogenicity, developmental — deal with an end effect, and we deal with the modality, the causative agent” [92]. Indeed, other than in the simplest cases, end effects were not known.

These four panelists then began to devise ways to explain why the product of their efforts would *not* prove satisfactory. Dr. Whittenberger advised that “whatever statement we make here will determine whether the agency can continue to use these interim guidelines. … [T]hat’s what they told me a few minutes ago. … If we say we think it’s adequate with modifications and should be used, that would keep them in business” [93].

The archival record of this 2-day meeting of the Complex Mixtures Panel nowhere recorded any participant contending that mixture toxicity was a myth, although Dr. Scala’s dismissal of the Love Canal experience came close. Nor did any participant contend that Superfund obligations could be met while ignoring complex mixtures, that current laboratory practices could meet the task posed even by simple mixtures, that Dr. Silbergeld’s excoriating review was fallacious, or that the guidelines, as then developing, would prove genuinely useful. Even so, no one spoke up against Dr. Scala’s criterion withholding from risk assessment any mixture unless the EPA already had data on more than a small fraction of its components.

Ambivalence was not confined to the Complex Mixtures Panel. That same spring, 1985, William Ruckelshaus, an attorney and a Department of Justice veteran and the EPA’s first and fifth administrator, newly resigned after his second successful EPA tour, wrote this: “During the past 15 years, there has been a shift in public emphasis from visible and demonstrable problems, such as smog from automobiles and raw sewage, to potential and largely invisible problems, such as the effects of low concentrations of toxic pollutants on human health” [36]. Risk assessment had arisen to make sense of such concerns, but risk *management* would now force a choice between avoiding harms as they might be demonstrated and avoiding harms as they might be anticipated. Among those not often demonstrated in the laboratory but increasingly anticipated in the public mind were “effects from chemicals in combination that are greater than would be expected from the sum effects of all chemicals acting independently” [36]. The second choice, the anticipatory choice, would require, ill-advisedly he thought, that “[a]ny identifiable risk ought to be eliminated up to the capacity of available technology to do so” [36]. He did not say, but he had to know, that the Benzene Decision put any “best available technology” rule out of reach, but, as a legal scholar, he would have appreciated three justices’ concurring hint about avoiding that precedent in future. The federal environmental-health establishment, most saliently the EPA, was indeed constrained by the Benzene Decision, but remaining constrained may have been less a necessity than a choice.

On 24 September 1986 in the *Federal Register*, the EPA released “Guidelines for the Health Risk Assessment of Chemical Mixtures” [94]. This document was, on the whole, reasonable, cautious, and modest, but reading it now with knowledge of its influence prompts several observations.

“Part A” presented the guidelines themselves. Forthrightly acknowledged was the complexity of most environmental mixtures. Yet the approach to complexity was not integrative — evaluating real mixtures in real environmental samples — but reductive to laboratory practice, evaluating minimized sets of chemicals in simplified ways, such as by assuming dose additivity and response additivity. Limitations were obvious, and warnings were clear — too clear for some advisors.

“Part B” was a response to comments. “Numerous comments were received concerning the assumption of additivity. … The Agency and its reviewers agree that as the number of compounds in the mixture increases, an assumption of additivity will become less reliable in estimating risk. … However, the Guidelines do explicitly state that as the number of compounds in the mixture increases, the uncertainty associated with the risk assessment is also likely to increase” [95]. Suggested here was the authors’ orientation not to the risk presented, such as to fish, by a complex environmental mixture, such as river water, but to the attribution of risk to one or two components of that mixture. “The Guidelines were further clarified to state that dose (or response) additivity is theoretically sound, and therefore best applied for assessing mixtures of similar acting components that do not interact” [95]. Neither form of additivity was inherently simplistic; at minimum, dose additivity could adjust for differences in potency, and response additivity could accommodate toxicants of different sorts. But advising that these methods were “best applied for assessing mixtures of similar acting components that do not interact” made them simplistic as policy guides and, in practice, unhelpful.

The isomer issue, as raised in the Seveso report, was not addressed. Had it been, then evaluating a mixture of two industrial pollutants could have meant evaluating a mixture of four, and four-at-a-time, while even three-at-a-time strained preferred mathematical methods. Toxicant interactions, such as synergy and competition for detoxifying metabolic pathways, were acknowledged but set aside as poorly described. Synergy — and toxicity itself except for chronicity and subchronicity and carcinogenicity — was assumed to be extinguished at low doses. So was synergism’s antithesis, antagonism. Special vulnerabilities of the fetus and the child were not considered. A technical support document, promised to placate Dr. Silbergeld, was missing; in its stead was a ten-sentence concluding section labeled “Need for a Technical Support Document” [94].

The complex-mixtures problem, brought home to the EPA by Superfund, pressed against the chemical-by-chemical methods that had made toxicology a successful laboratory science.

A toxicant exposure could be characterized exquisitely in laboratory animals if studied carefully enough: one route, one intensity, one frequency, with individual animals as similar to each other as possible. Routes, intensities, and frequencies could then be varied in different cohorts. Such studies might have been prompted by epidemiological observations but would be experimental, not observational, not blurred by avoidable confounders. Similar studies could also function prospectively to screen chemicals, each one individually, for toxic effects in animals — or in Ames-test bacteria or in cell lines — and, by extension, in humans.

Toxicological methods had been developed to test single chemicals. Testing two or more or many chemicals in various mixing ratios or testing actual environmental samples would require, at best, methodological elaboration and would initiate, at worst, methodological drift, a loss of procedural standardization. With prospective mixture studies inherently subject to challenge for their selection of chemicals, for the ratios at which selected chemicals would be mixed, and for the targets exposed [96], generalizability would be hard to achieve. All that conceded, methods for in vivo toxicity testing of complex mixtures, including environmental samples, were not unavailable; the National Research Council published a book about them in 1988 [97].

Remaining to be posted are three advisories.

First, reluctance has not been recalcitrance. For example, as guided since 1972 by the Clean Water Act [98], the EPA has routinely assessed “whole-effluent toxicity” (WET) as a function of organismal response [99]. Plainly, though, effluents — liquids flowing from pipes — have not been the only environmental media whose organismal response could be assessed.

Second, a presentist view may look back unfairly. Scientific insights and technical advances in the later years of our study period made knowable what had been hard to know, or impossible to know, in the earlier years. Notably, the “omics” revolution — genomics, transcriptomics, proteomics, metabolomics — has expanded not only the questions we can answer, whether directly or indirectly, but also the questions we can imagine asking. The path from exposure to biological response to pathophysiology has long been followed, but following has become easier, not just in individual patients but also in communities and not just for individual toxicants but also for the mixtures conveyed in environmental media. Toxicant exposures, from simple to complex, create metabolic profiles, which can suggest mechanisms of injury. Investigators studying environmental mixtures can now more easily compare metabolic profiles between and among individuals, groups, and communities differing in their exposures, such as chronic exposures to ultrafine particles at different distances from highways [100].

Third, even if toxicologists with regulatory influence had not feared methodological drift but had followed logic and evidence to the study of risk in all its complexity, the constraints, statutory and judicial, within whose boundaries they worked would have kept their findings from altering regulatory policy.

### Guidelines reconsidered

Ellen Silbergeld, PhD, went on to become Professor of Epidemiology, Environmental Health Sciences, and Health Policy and Management at the Johns Hopkins University Bloomberg School of Public Health, author or co-author of roughly 500 scholarly publications, and recipient of, *inter alia*, a lifetime achievement award from the Society of Toxicology and a “Genius” award from the MacArthur Foundation. On 6 June 2017 we met in College Park with Dr. Silbergeld for over 2 h. She asked to be named and cited.

Dr. Silbergeld was sensitive to the predicament facing United States regulators, constrained as they were by statutes authorizing the regulation of individual chemicals but effectively precluding the regulation of mixtures. Science, she said, had failed to give regulators the technical information they needed; her insistence that the original EPA guidelines include a technical report had been well placed. “I think I didn’t have quite the appreciation I’ve gained [since then] at how limited toxicology is, and I got that from really understanding systematic methods. And systematic methods are basically a form of critical thinking which has never really been applied in toxicology”. Recently she had second-authored a critical review of toxicology, comparing it unfavorably to evidence-based medicine [101]. That said, and that written, she attributed regulation’s current difficulties in part to external pressure. Recalling early experiences in advocacy, she noted that “[d]uring my time at EDF [the Environmental Defense Fund], I saw the chemical industry identify ‘mechanism’ as their new tactic. Their hope [was] that we wouldn’t be able to figure out the mechanism for most things. … These agencies get set up by stakeholders, and they [the stakeholders] want to be in control of it [the regulatory process]”.

She did not wish to consider the environmental interaction of many thousands of industrial chemicals, calling that image “a googolplex”, googol being 10 raised to the 100th power. She preferred to consider a much smaller number of intensively produced chemicals but was still resistant to considering their interaction, since no regulatory response was conceivable.

The EPA’s initiatives under a cumulative-risk-assessment heading were less constrained than under a mixture-toxicity heading because “cumulative” risks, all of dissimilar sorts, were not “accumulated” risks, all of similar sorts. Being all of *dissimilar* sorts, even incompatible sorts, cumulative risks did not prompt the additive toxicological procedures applied to chemical mixtures — whose regulation, she insisted, current law in the United States, and in other countries, too, simply would not allow. “To regulate mixtures, the *law* must be changed”.

Why had EPA and other responsible agencies not tried to educate the public about mixture toxicity and its putative implications? Because they could not do that, nor could they lobby Congress. They could not initiate contact in order to educate or persuade. They could only try to wrangle an invitation to testify at a hearing or in some other way inform Congressional staffers.

Are we looking through the wrong end of the telescope? Was regulatory futility not so much the challenge as it was the message? Rather than obsessing about individual chemicals analyzed outside their commercial formulations or environmental contexts, should we instead start planning a long careful transition to closed-loop — or zero-toxicant-emission — industrial practices? Yes, with inside-the-plant safety first assured, probably.

### Cumulative risk conceded

Community health advocates in the 1980s had begun urging the EPA and state environmental agencies to address toxicants’ cumulative effects on disadvantaged populations. Some advocates became activists, first charging “environmental racism”, then demanding “environmental justice”, and eventually reconceptualizing risk assessment [102–104]. They proceeded holistically, examining ignored communities, their socioeconomic predicaments, their health problems, and their surrounding industries [105]. The EPA never proceeded in this way. William Ruckelshaus knew its failure to do so was a weakness, and, following his second term as administrator, he called for legislation to add such activities to the agency’s mandate [36].

The federal government was not deaf to these concerns. From Congress in 1988 came a request to the National Academy of Sciences to characterize whatever risks might be posed by pesticide residues consumed in early life. In 1992, President George H. W. Bush established the Office of Environmental Justice in the EPA. In 1993, the National Environmental Justice Advisory Council (NEJAC) began operating as a formal advisory committee chartered to advise the EPA administrator [106].

Also in 1993, in response to the Congressional request made 5 years prior, the NRC published *Pesticides in the Diets of Infants and Children*, its writing committee chaired by a pediatrician-toxicologist, Philip J. Landrigan, MD, MSc, Mount Sinai School of Medicine, New York City [107]. Standard risk assessment had not adequately appreciated how often and in what ways children “may be exposed to multiple pesticides with a common toxic effect” [108] and had too often ignored “additive and synergistic effects” [109] and the potential for pesticides to be “converted in the environment or in vivo to form metabolites with toxicity potentially greater than that of the parent compounds” [110]. Moreover, routinization of the childhood diet brought into consideration “cumulative exposure” [111], which was more typically categorized as an occupational issue. Especially perceptive were discussions of low-level synergism and the toxicity of “inert ingredients” [112].

In 1994 President Bill Clinton issued Executive Order 12,898, declaring that “each Federal agency shall make achieving environmental justice part of its mission by identifying and addressing, as appropriate, disproportionately high and adverse human health or environmental effects of its programs, policies, and activities on minority populations and low-income populations”. The administrator of the EPA was to convene an interagency Federal Working Group on Environmental Justice and, among other tasks, “conduct environmental human health analyses [which], whenever practicable and appropriate, shall identify multiple and cumulative exposures” [113]. Also in 1994, the National Research Council published *Science and Judgment in Risk Assessment*. Therein the NRC urged the EPA to reconsider its routines: “Some experts have noted that important aspects of risk are neglected by EPA. The agency does not appear to recognize the possibility of synergistic interactions when multiple chemical exposures occur, nor does it seem concerned that available data show extreme variability among individuals in their responses to toxic substances. The failure to deal with those issues can lead to serious underestimation of human risk, especially at very low exposures. A related issue is the overlooked problem of risk aggregation — how risks associated with multiple chemicals are to be combined” [114].

In August 1996 came two new statutes assigned for administration to the EPA, the first one suspect in origin and equivocal in effect.

Back in 1993, the Natural Resources Defense Council, the NRDC, through successful litigation, had forced the EPA to end its lax, or *de minimis*, enforcement of two provisions of the Food Additives Amendment of 1958, which had strengthened the Federal Food, Drug, and Cosmetic Act of 1938. The provisions — which became famous jointly as the “Delaney Clause”, after their author, Congressman James Delaney of New York — were these:

“(3) No such regulation [prescribing safe use] shall issue if a fair evaluation of the data before the Secretary —

- “(A) fails to establish that the proposed use of the food additive, under the conditions of use to be specified in the regulation, will be safe: *Provided*, That no additive shall be deemed to be safe if it is found to induce cancer when ingested by man or animal, or if it is found, after tests which are appropriate for the evaluation of the safety of food additives, to induce cancer in man or animal; or
- “(B) shows that the proposed use of the additive would promote deception of the consumer in violation of this Act or would otherwise result in adulteration or in misbranding of food within the meaning of this Act [115].

Since the term “food additive” had been defined to include “a pesticide chemical”, strict enforcement of the Delaney Clause made legislative relief a priority for the pesticide industry, which lobbied vigorously for what would become the Food Quality Protection Act of 1996, the FQPA.

A more presentable prompt for the FQPA had been *Pesticides in the Diets of Infants and Children*, but on 22 May 1996 Dr. Landrigan himself opposed the bill as then written, noting that it required the recommendations of his report to be implemented but then violated its own requirement, in part by removing pesticides from scrutiny under the Delaney Clause. Dr. Landrigan, testifying about the bill’s Senate version, went on to castigate a provision allowing:

that a pesticide standard need not be health based if the imposition of a health-based standard would make unavailable a pesticide that is needed to maintain a particular food crop, “taking into account national and regional effects”. This small paragraph is a classic example of an exemption that eats a rule. … Mr. Chairman, as a pediatrician, I find this position unacceptable. … The incidence of cancer in America’s children is increasing. … With all due respect, I submit that S. 1166 is bad legislation. … I urge you not to enact this legislation [116].

The FQPA as finalized, and as passed unanimously by both houses of Congress in August, did not contain the language Dr. Landrigan had opposed, but it remained at best a compromise. However, it did require the EPA to assess the dietary risks posed to fetuses, infants, and children by the “cumulative effects” of pesticides having “a common mechanism of toxicity” and by “aggregate exposures” to any particular pesticide — unfortunately applying the terms “cumulative” and “aggregate” contrary to expected usage [117]. The FQPA rewrote the regulation of pesticides, extensively amending FIFRA and the Federal Food, Drug, and Cosmetic Act to do so, and in its Title IV effectively removed pesticides from Delaney Clause jurisdiction and elaborated a new system of tolerances and exemptions for pesticide chemical residues. A “pesticide chemical”, singular, comprised “all active and inert ingredients”, plural. The word “inert” appeared in that one phrase and nowhere else [118]. The only ingredient to which the regulation of a pesticide chemical was to apply would still be the single chemical declared “active” by the manufacturer. Moreover, any distinction between “active” and “inert” was to be guarded as a trade secret and thus would not be shared with the public: “Data and information … shall be entitled to confidential treatment for reasons of business confidentiality” [119]. The FQPA ignored synergy, mentioning mixtures substantively only in reference to nitrogen stabilizers; when in a mixture with fertilizers a nitrogen stabilizer had to be labeled a commercial product’s sole active ingredient [120]. With pesticide formulations once again not to be evaluated as mixtures, Dr. Spurrier’s 11-year-old hypothesis — the EPA would not study pesticides as mixtures even if allowed to [71] — would remain untested.

Singularly resented in the FQPA was that two or more toxicants encountered through individually allowable exposures would be recognized as potentially dangerous only if they shared a “common mechanism of toxicity” [121]. Here in this common-mechanism stipulation was “additivity” again, and the environmental-justice movement was not willing to accept this precondition. Mechanisms of toxicity were not just “common”. They were complex: chemical stressors, yes, but social stressors, too, however understudied and however out-of-place they would seem to be in the company of chemicals [105].

Three days later came the Safe Drinking Water Act Amendments of 1996, the SDWAA, the second new statute assigned for administration to the EPA. Despite having been marked-up and enacted in parallel with the FQPA, this was unambiguously a different bill: “The [EPA] Administrator shall conduct biomedical studies to … develop new approaches to the study of complex mixtures, such as mixtures found in drinking water, especially to determine the prospects for synergistic or antagonistic interactions that may affect the shape of the dose–response relationship of the individual chemicals and microbes, and to examine noncancer endpoints and infectious diseases, and susceptible individuals and subpopulations”. Moreover, studies were to take “into account in the case of pesticides the time of application of the pesticide for the source water area and the travel time for the pesticide to reach such waters” [122].

However discordant their mandates, these new laws brought complementary possibilities. One required that cumulative risk be assessed, and the other required that complex mixtures be taken seriously. The EPA, in the environmental-justice movement’s view, now had its orders: catch on and catch up.

In 1997, the EPA administrator, Carol Browner, acknowledged that assessments had to account for combined effects. She announced that efforts were underway to develop methods for cumulative risk assessment and that these efforts would lead to new guidelines. But she also cautioned about stretching quantitative risk assessments to include social stressors: “While we can more consistently take into account many new factors in this approach to risk assessment, many other potentially important factors are more difficult to include in our analyses, particularly the social, economic, behavioral or psychological factors that also may contribute to adverse health effects. … Assessment of these factors is often hampered by a lack of data to establish plausible cause-and-effect relationships; difficulties in measuring exposure, incidence and susceptibilities related to these risks; and few methods for assessing or managing these risks. This guidance does not address these factors” [123]. The tension was real. The EPA already had many laws to obey and many different specific analyses to perform and was now being required by the FQPA and the SDWAA to add more. Pointedly, the EPA would conform to the FQPA’s cumulative-risk instruction but would not conform to activists’ pressure to incorporate adjunctive factors of whose suitability for toxicological study it was as yet unconvinced. The case for nonchemical stressors, Browner felt, had not been made [124].

However awkward the moment, planning and “scoping” began immediately [125]. Five years later, 2002, came *Guidance on Cumulative Risk Assessment of Pesticide Chemicals That Have a Common Mechanism of Toxicity*. The EPA had not caught on, explaining that “[a] cumulative risk assessment begins with the identification of a group of chemicals, a common mechanism group (CMG), that induce a common toxic effect by a common mechanism of toxicity” [126]. Additivity was still a precondition, headway on mixtures was minimal, and synergism was mentioned just twice, both times tangentially.

The next year, 2003, EPA published the *Framework for Cumulative Risk Assessment*, a document written to promote consistency by defining terms. It did not succeed in doing so. Following the FQPA [117] and still confusing, “aggregated” risks were repeated single-agent or single-stressor exposures, while “cumulative” risks were multiple-stressor exposures, such as to different pesticides but of the same sort, so as to preserve additivity. The *Framework* did allow qualitative analysis when data were limited but otherwise stuck to familiar routines. The EPA now defined cumulative-risk assessment as “an analysis, characterization, and possible quantification of the combined risks to health or the environment from multiple agents or stressors” [127], but this definition was predicated on the four Red Book [59] steps — hazard identification, dose–response assessment, exposure assessment, and risk characterization — taken to vet single chemicals. After acknowledging the NRC’s admonition from 9 years before, the *Framework* was quick to preempt high expectations: “As of August 1, 2001, there were 19,533 pesticide products on the market … and 79,120 existing chemicals on the Toxic Substances Control Act inventory …. Each year, a number of chemicals are added. Assessing the cumulative effect of these chemicals will be a great challenge to to [*sic*] the field of risk assessment and to the Agency”. Paradoxically, though, the document acknowledged that cumulative-risk assessments were already being performed successfully in ecological settings and in “tribal cultures” and that “[t]he framework [for cumulative risk assessment] itself is conceptually similar to the approach used in both human health and ecological assessments, but it is distinctive in several areas”. Contrary to the NRC view, the *Framework* dismissed the terms “*synergism*” and “*antagonism*,” saying, “These terms are only marginally useful, in part because the underlying toxicologic concepts are only defined for two-chemical mixtures, and most environmental and occupational exposures are to mixtures of many more chemicals” [127]. This last sentence, no doubt unintentionally, implied that toxicological concepts were inapplicable where most needed.

Discouragement deepened, as standard practices seemed sure to mischaracterize the totality of cumulative health risks associated with actual exposures to diverse and dynamic combinations of chemical stressors — and, putatively, nonchemical stressors, too. Laboratory toxicology could not be expected to succeed at an integrative task, an environmental-ecological-epidemiological task. Yet laboratory toxicology remained the empirical arbiter of toxicant regulation, although it had little to contribute to the study of human nonchemical stress even if mediated physiologically through the autonomic nervous system and the hypothalamic–pituitary–adrenal axis and even if shown to accentuate chemical stress itself.

In December 2004, NEJAC reported, at length, on cumulative risks and impacts [2]. Much remained tentative, with a search still on for cumulation criteria intuitively sensible and experimentally verifiable [128–131].

The EPA was still not catching up. *Phthalates and Cumulative Risk Assessment: The Tasks Ahead*, a 2008 NRC report sponsored by the EPA, was blunt: “In cumulative risk assessments of human health effects, there is a reliance on dose addition as the default approach. Current practices focus on well-defined mixtures of chemical stressors to which simultaneous (or concurrent) exposures occur. … [A]lthough multiple methods are available, EPA has used only a few of them in practice. And despite recognition of nonchemical stressors as potentially important contributors to cumulative risk, nonchemical stressors are rarely addressed or evaluated” [132].

Regulatory responsibility for environmental toxicants rested principally with the EPA. And “rested” was a verb fairly applied in some respects. To its credit, the EPA knew this and again sought advice from the National Research Council, which in response this time formed the Committee on Improving Risk Analysis Approaches Used by the U.S. EPA. In February 2009, the Committee published a 403-page report, *Science and Decisions: Advancing Risk Assessment*, soon nicknamed the “Silver Book” [133]. The Committee found much to fault.

Risk assessment practices had fallen short of the EPA’s own intentions. Nonchemical stressors had not been seriously considered, nor had person-to-person or community-to-community variation in vulnerabilities. Ecological risk assessment, in which the EPA was deeply experienced, employed techniques applicable to human-community risk assessment but had rarely been used in human communities. Interactions between chemical and nonchemical stressors had not often been studied in ways informed by epidemiological, physiological, or pharmacokinetic modeling [134]. Ensuring protection in, and of, the environment required sensitivity to signals of potential harm, not only characterization of identified risks [135]. Assessment of low-dose risk — whether eliciting a linear response or a nonlinear response — had shown “substantial deficiencies” [136]. A focus on single-chemical carcinogens — and therefore on “complete” carcinogens — had critically underappreciated cancer’s multifactorial nature [137]. Animal data had not, and could not, reliably address human heterogeneity, whose appreciation was profoundly important when characterizing the risk posed by any toxicant [138]. EPA’s cumulative-risk assessment paradigm had not credibly considered nonchemical stressors or synergism or antagonism or community vulnerability or multiplicity in routes of exposure [139].

Appendix E of *Science and Decisions* recorded the Agency’s responses. Two were notably concessive. (1) “Our ability to evaluate mixtures and potential interactions (other than that provided under EPA’s current mixtures guidance) is limited” [140]. (2) “Whole-mixture studies are routinely used in ecological risk assessments” [141].

Despite the NRC’s 2008 and 2009 admonitions to the EPA, the real-world challenge the environmental-justice movement most wanted addressed — the interaction of chemical stressors and nonchemical stressors, both of which were disproportionately high in disadvantaged communities [142] — had not attracted adequate investigational interest.

All the while, though, evidence supporting the importance of cumulative-risk assessment was mounting. As Dr. Silbergeld had envisioned in her 1984 critique [70], toxicants *not* sharing a “common mechanism of toxicity” were more regularly being seen to exert cumulative effects. For example, dosing pregnant rats with mixtures of seven [143] — and, in a later study, ten [144] — antiandrogens not all sharing a common mechanism of toxicity would elicit cumulative teratogenic effects in male pups; the effects would exceed in severity what would be expected if each chemical had been acting individually, one after another, rather than together. A review citing work published no later than 2016 found abundant evidence of antiandrogens causing harm cumulatively and found that dose addition and response addition and “integrated” addition, by which the prior two additions were merged mathematically, could each play methodological roles when evaluating antiandrogenic chemicals, whatever their mechanism, at least when acting on androgen-dependent reproductive development [145].

The cumulative corollary as presented by NEJAC was necessarily, and knowingly, disruptive [64, 146, 147]. Chemical toxicants and nonchemical stressors might compound each other’s effects, but the nonchemical stressors nominated included ones less toxicological than sociological or psychological, such as poverty and adverse childhood experiences (ACEs) [148]. Yet others, while still related to poverty and adversity, were reassuringly pathophysiological. Malnutrition would encompass many examples. For instance, disadvantaged communities have characteristically been dependent on agro-industrially produced, processed, packaged, and purveyed foods, with which chemical toxicants have long been associated [149]. As reported during our study period, women with higher risks of toxicant carriage have disproportionately been members of disadvantaged communities [19].

Following a second rationale, nonchemical stressors, such as anticipation of violence, if “dosed” chronically, might prove cumulative by increasing “allostatic load”, upsetting homeostasis, maintaining a hormonal stress response [150, 151], and predisposing to arterial hypertension and its sequelae [3, 133, 152–154]. Empirically, maternal stress in a rodent model had been found to worsen the effects of gestational lead exposure in offspring [155], and similar results had been found for methylmercury [156].

Following a third rationale, chemical-stressor exposures made more likely by poverty and adversity might cause genetic mutations. Or they might cause epigenetic changes, which alter the activity of genes — such as by turning them “on” or “off” — without altering genes themselves. Epigenetic changes are not mutations but, within limits, are heritable.

That last possibility was an epiphany. While required for the timing and regulation of normal growth, development, and function, epigenetic changes could be disadvantageous and, when occurring in germ-line cells, could harm as many as three future generations [31, 32, 157]. This transgenerational feature suggested — did not prove but legitimately suggested — that adversity might, to some degree, be heritable biologically as well as socioeconomically. Environmental-justice activism and cumulative-risk activism began to coalesce.

NEJAC recommended actions to “incorporate social, economic, cultural, and community health factors, particularly those involving vulnerability, in EPA decision-making … and … to reduce cumulative risks and impacts in disadvantaged, underserved and environmentally overburdened communities through community-based and collaborative approaches” [2]. NEJAC knew that for poor communities environmental risk was inescapably cumulative, and the *Framework’s* concurrence, however provisional, marked “the maturation of environmental justice issues” first brought to national prominence a generation before [2]. NEJAC had reason to feel vindicated, even optimistic, but frustrations would prevail.

### Sensitivities observed

We knew the EPA had repositioned its mixture-toxicity program within its cumulative-risk program but did not know why. The mixture-toxicity program had been attracting criticism for failure to engage complexity, but the cumulative-risk program had been attracting criticism for failure to engage nonchemical stressors. Neither was proceeding smoothly, but the cumulative-risk program did have comparative advantages. It had a focussed and vocal constituency, the environmental-justice movement, and it had evidence of community-scale harm, assuming “the social determinants of health” [158] included nonchemical stressors.

To help us interpret these observations, we interviewed an active senior EPA officer privately and confidentially outside EPA headquarters 12 May 2015. Our questions were precise. The officer’s answers were laconic.

Had mixture toxicity been “walled off” within the EPA? Not consciously. Was mixture toxicity thought impossible to assess? No, but EPA had no capacity to assess it well. Was mixture toxicity thought impossible to ameliorate? Yes. Or regulate? Yes. Was it politically toxic? Yes.

Had mixture toxicity been subordinated to cumulative-risk assessment (CRA)? Mixture toxicity had been placed in the “Special Occasion” box [not ordinarily to be opened] while environmental justice (EJ) was being pushed out of CRA under a new director — ironically, since EJ had been the “CRA champion”.

Had a risk-assessment methods emphasis, which had impeded risk-management action on mixtures toxicity and cumulative risk, co-opted scientific critics? Maybe. Were scientists taking refuge in methods refinement? No. Politicians were.

Had CRA been designed to fail? No. Had CRA been designed to placate the EJ lobby? Yes. Had CRA been designed to occupy social scientists? No. Did CRA’s mechanistic diversity exculpate the chemical industry by diffusing liability into ambiguous interactions of chemical and nonchemical factors? Maybe.

Regarding CRA and EJ, were CERCLA, and the EPA generally, limited by their enabling statutes? Maybe.

### Stasis sustained and escaped

While it did not change the definition of “mixture” or remove statutory constraints on the EPA’s ability to evaluate mixtures, the Frank R. Lautenberg Chemical Safety for the 21st Century Act of 2016 [159] — a long awaited bundle of amendments to TSCA — did specify additional testing authority and alternative testing methods, meaning methods less dependent on vertebrate animals, and it did finally establish a program, replete with requirements and deadlines, to evaluate all chemicals currently in use and to ban chemicals or reject new ones on findings of excessive risk, with risk–benefit claims still to be entertained but less likely to be dispositive. However, the distinction between active and “inert” ingredients — now active and “inactive” — remained, yet, unlike the FQPA, the Lautenberg Act required manufacturers to apply for the privilege of masking an “inactive” ingredient’s identity. Some new wording hinted at the obvious, that commercial formulations were mixtures, but routine toxicological assessment of commercial formulations rather than only their declared active ingredients was still not required — or mentioned. Much that the EPA now could do and much that it now had to do could not be done quickly. Some delays had been built in, and some would be inevitable. Crucially, evaluating all chemicals currently in use, or even just the yearly average of new chemicals for which a Premanufacture Notice would be filed, was far beyond the agency’s capacity [160].

All that said, the Lautenberg Act did open a path to environmental justice, at least along a chemical-stressor cumulative-risk dimension. Aggregate exposures and “the likely duration, intensity, frequency, and number of exposures” were to be considered [161], and “potentially exposed or susceptible subpopulations” were henceforth to receive particular attention. This new term, “potentially exposed or susceptible subpopulations”, appearing 18 times, meant “a group of individuals within the general population identified by the Administrator who, due to either greater susceptibility or greater exposure, may be at greater risk than the general population of adverse health effects from exposure to a chemical substance or mixture, such as infants, children, pregnant women, workers, or the elderly” [162]. This language did not refer to disadvantaged communities, even obliquely, but groups of individuals at greater risk may be found, perhaps disproportionately, in those communities [163]. An EPA administrator determined to advance environmental justice could now more easily find a statutory way forward.

Other units of the federal environmental-health establishment took their own paths. Instructively contrasting were the attitudes of the ATSDR and the NIEHS.

The ATSDR — the Agency for Toxic Substances and Disease Registry, administered by the Director of the CDC — issued mixtures statements in 2004 and 2005 and then in February 2018, after our study period, released *Framework for Assessing Health Impacts of Multiple Chemicals and Other Stressors (Update)* [164]. This 2018 document avoided the terms “synergism” and “antagonism”, which concepts the ATSDR attributed to experimental uncertainty [165]. The authors advised that “[t]he use of ‘greater-than-additive’ is preferred over the use of the term synergism … [and] ‘less-than-additive’ is preferred over the use of the term antagonism” [166]. The ATSDR authors seemed unconcerned that safety testing of industrial chemicals had been negligent [167], especially when granting “conditional registration” for pesticides [168], that safety testing of marketed formulations had been rare [169], and that commercial pesticide formulations containing ostensibly inert (or, from 2016, “inactive”) ingredients could be orders-of-magnitude more toxic than their declared active principal ingredients [170, 171].

Beyond seeming to be unconcerned, the ATSDR authors seemed to be unaware that the pesticide industry, by 2018, had already been shown to know that synergism within its formulations was real, that it was intentional, and that it was biocidal. In July 2016 an intensive search of applications for patents on pesticide formulations had reported that 96 of 140 (69 percent) had been described by their respective manufacturers as demonstrating ingredient synergies. These synergies, rather than going unmentioned out of fear that their documentation would increase regulatory scrutiny, were being presented to strengthen claims of novelty and, hence, patentability [172]. A year later, on 31 July 2017, internal documents — 75 e-mails and their threads — were finally made public by court order during litigation concerning Monsanto’s (now Bayer’s) Roundup® herbicide, whose declared active principal ingredient, its toxicologically vetted and regulated component, was glyphosate. These messages said, *inter alia*, that “the formulated product (and thus the surfactant) does the damage” [173] and that “surfactant in the formulation will come up in the tumor promotion skin study because we think it played a role there” [174]. The focus in these comments was not glyphosate. The focus was the formulated product, which included ingredients legally classed as inactive yet credited, in the surfactant’s case, as pivotal to herbicidal performance and, worryingly, to tumor promotion in a skin study performed, presumably, in nonhuman animals. Glyphosate’s effect, then, was known to be enhanced by co-formulation. Put differently, Roundup’s effect was known to be enhanced by synergism. Darkening this Monsanto story had been other e-mails prompting the EPA’s Inspector General to suspect collusion [175].

A more hopeful story may be told of a different unit.

In 2013 the National Institute of Environmental Health Sciences, the NIEHS, pointed proudly to a mixtures-science record begun 30 years before, in 1983, with a study first-authored by the Institute’s eventual director, Linda Birnbaum, PhD [176]. The study had shown that a troublesome flame retardant “[p]reviously reported to be a single compound” was instead “a mixture of two closely related isomers” differing in toxicity [177]. Birnbaum’s discovery should have compelled the EPA’s Complex Mixtures Panel to take isomers into account but did not. Her discovery might also have sparked a high-priority mixtures interest at the NIEHS proper but did not do that either.

In 1986, though, the Institute acquired an in-house mixtures interest when the Superfund Research Program (SRP) — formally the NIEHS Hazardous Substance Basic Research and Training Program — was established. Through extramural funding the SRP would support research bearing upon Superfund sites and, thus, bearing upon complex mixtures as encountered in the field and as modeled in the laboratory. Still, the NIEHS issued no “Request for Applications” (RFA) in the mixtures-toxicity or cumulative-risk fields until 1997 [176].

In 2009, after 19 years at the EPA, Dr. Birnbaum was named director of the NIEHS and its National Toxicology Program, the NTP, vowing “to create a holistic approach that can deal with the biggies, from complex mixtures of toxic chemicals to climate change” [178].

In 2011, a gathering called “The NIEHS Mixtures Workshop” signaled a new emphasis [176], and in August 2012 the Institute published its strategic plan for the next 5 years, 2012–2017. The plan veered sharply from the EPA line. “NIEHS endeavors to support environmental justice research, by defining the environmental factors and their complex interactions that contribute to environmental health disparities, and by studying chemical and nonchemical stressors at the community level”. Also freshly on-topic were “the economic impacts of environmental health risks, decisions, and policies”. A major goal now was understanding “how combined environmental exposures affect disease pathogenesis”. Not whether they do but how they do, such as epigenetically. The more provocative words “mixtures”, “cumulative”, “synergy”, and “synergism” nowhere appeared [179]. EPA’s reluctance to study more than two mixed toxicants, but then only similar ones, was now being set against the Institute’s commitment to study whatever needed to be understood: the “exposome” [180], meaning the totality of an individual’s exposures from before conception onwards through the lifespan, plus the microbiome plus the genetic and epigenetic diversity of human vulnerability [179]. Exposome research would be maximally integrative. It would presuppose mixing in vivo and could implicate individual and co-occurring toxicants by associating them with harm in straightforward clinical-pathological fashion.

By 2013 a review of mixtures research at the NIEHS showed a gratifying variety: “Some examples of the types of mixtures studied include: groundwater contaminants, pesticides/fertilizers, dioxin-like chemicals (assessing the toxic equivalency approach), drug combinations, air pollution, metals, polycyclic aromatic hydrocarbons, technical mixtures (e.g. pentachlorophenol, flame retardants), and mixed entities (e.g. herbals, asbestos)” [181]. A mixtures-in-epidemiology conference followed in 2015 [182], as did a low-dose-theory symposium focused on the Halifax Project [33]. Dr. Birnbaum began this last listed event by declaring “The toxicology of the twenty-first century should not be the toxicology of the twentieth century” [183]. Further events would follow [184].

Finalized in October 2016 was an ambitious new multidisciplinary research concept the NIEHS called PRIME, for “Powering Research through Innovative Methods for mixtures in Epidemiology” [185]. Applications for PRIME grants were reviewed beginning 22 January 2017 [186], and many were funded.

All that said and done, the NIEHS was built for investigation, not regulation. NIEHS could affect policy only with scientific evidence, not with legal authority, and was still not of one mind. As late as 27 April 2017, an NIEHS webinar on complex environmental mixtures opened with the EPA’s 1986 *Guidelines*. Progress since 1986 was being described mostly as refinement in additivity: modernizing the search for pollutants exhibiting common behaviors so as to reduce the dimensions of whatever statistical model was to be estimated [187].

The next NIEHS strategic plan, for 2018–2023, would be shorter, less expansive, and more cautious, with “mixtures” snuck back in only to explain the meaning of “combined exposures” [188]. Dr. Birnbaum would relinquish her directorships, but not her research, in 2019 [189]. She would not have resigned, she would explain in February 2021, had she not grown “tired of being so tightly controlled” [190].

### Stasis interpreted

Why had the federal environmental-health establishment for so long been so reluctant to correct its perspective? We asked Thomas A. Burke, PhD, MPH, the Jacob I. and Irene B. Fabrikant Professor and Chair in Health Risk and Society in the Department of Health Policy and Management at the Johns Hopkins University Bloomberg School of Public Health. Dr. Burke is jointly appointed in the Department of Environmental Health Sciences and the Department of Oncology in the School of Medicine and directs the Johns Hopkins Risk Sciences and Public Policy Institute. He chaired the NRC’s Committee on Improving Risk Analysis Approaches Used by the U.S. EPA and supervised the writing of *Science and Decisions*, the “Silver Book”. He was nominated by President Barack Obama to serve as EPA Assistant Administrator for the Office of Research and Development and from January 2015 until January 2017 was the EPA’s Science Advisor and its Deputy Assistant Administrator for Research and Development. The United States Senate never voted on Dr. Burke’s nomination; he served without confirmation and resigned upon change of administrations. We met with Dr. Burke in his office 18 June 2019 for an hour and a quarter along with his Hopkins colleague, Mary Fox, PhD, MPH, an expert in environmental mixture toxicity, its putative relationship to racial and ethnic health disparities, and cumulative risk overall.

“There’s enormous concern about cumulative risk in general from industry”, Dr. Burke said, “[and] there always has been”. The agricultural-chemical industry exhibited a “dread of epidemiology”, which was “really the only science that addresses cumulative risk”, meaning that toxicology did not, “and was detecting risks — say, for non-Hodgkin’s lymphoma related to Roundup or glyphosate — that might be really an indication of true elevation of risk in workers that are exposed to a really complicated mixture [a declared active principal ingredient plus its “inactive” co-formulants] and have the other social factors and healthcare factors that put them at increased risk. … There was tangible anxiety about … really difficult regulatory implications. … I can tell you virtually every major issue I dealt with at EPA was a cumulative-risk issue. … I would have to say from a science perspective I felt very good that cumulative [risk] — not the social aspects of risk but from a chemical perspective — [was included in] the problem definition [of] almost every major issue. Now, frankly, that doesn’t mesh well with the regulatory mandates of the agency, so that makes it difficult because the regulatory mandates are very substance-specific, agent-specific. … The statutes [establishing the EPA’s regulatory mandates] are the antithesis of cumulative risk”.

What would Dr. Burke do if political circumstances ever favored concerted action?

He would overhaul TSCA, even as revised in 2016 by the Lautenberg Act. Senator Lautenberg died in office in 2013, and his namesake act was passed in a form equivocal with respect to mixtures and, more broadly, cumulative risk. And, in effect, it prohibited state-level leadership.

Dr. Burke would shift safety’s “burden of proof” from the EPA to industry. He would rehabilitate the EPA’s original “best available technology” engineering mandate and deemphasize its “risk assessment” toxicology mandate, the latter dominant since 1983 but easily impeded legalistically. He would require “systems thinking”, such as he himself has facilitated by empaneling the Environmental Health Matters Initiative, the EHMI, within the National Academy of Sciences.

Dr. Burke noted that public education has not heretofore been in the EPA’s mandate, but he thought it could be put there. “It [leadership, including public education] comes from the top [meaning the presidency] and [would require] a fully functioning science infrastructure [including the CDC] that has a strong public-health component to make it work”. Decisive action, such as a transition to closed-loop industrial production and non-production of egregious toxicants, “is way bigger than a regulatory agency”.

## Conclusions

The United States federal environmental-health establishment had already lost much of its ability to respond effectively to the harms studied here before it ever thought to do so.

Congress had defined pesticide formulations, which were chemical mixtures, as single entities and had limited regulators’ examinations to only those ingredients declared to be “active”. It had defined any chemical mixture restrictively, excluding from study environmental mixtures reacting to form “new” chemical substances. And it had allowed any mixture to be assessed simply by characterizing, or referencing past characterizations of, its individual components, unless top management directed otherwise.

The Supreme Court complicated matters for environmental regulators — but also simplified matters for them — by striking down OSHA’s inadequately explained requirement that polluters reduce their emissions to the lowest technologically feasible levels they could afford. Conscientious regulation of no-safe-dose toxicants became harder to envision than it would otherwise have been, when regulating toward zero was disallowed. For whatever reasons, the environmental-health establishment never did formulate a new standard that avoided OSHA’s past errors, despite what seemed a reasonable chance of success under review by a future court.

These constraints, statutory and judicial, had come into place five months before Superfund was created. Recognizing an obligation to respond scientifically to the challenge presented by management of sites contaminated by complex mixtures, the EPA convened a group to consider mixture toxicity and write guidelines for assessment of risk. But the EPA had already grown accustomed to its constraints and showed little interest in escaping them, evidently because constraints were also now internal, even self-imposed. Efforts to write guidelines for evaluating mixture toxicity and later for evaluating cumulative risk displayed a fear that toxicology’s methods would be set adrift by requiring the assessment of multiple toxicants mixed in multiple ratios encountered in multiple exposures. Even if assessment were to venture beyond single chemicals, regulation seemed sure to be futile if any chemical’s safety were seen as contingent upon the presence or absence of some number of other chemicals in various amounts. Assessing and regulating chemicals one-by-one was hard enough; assessing and then regulating chemicals in mixtures promised to be exponentially harder, even impossible without reconceptualization of both regulatory mission and strategy.

Embracing complexity would have required the EPA to sponsor research questioning its own practices and disquieting industries adapted to those practices. Yet one other unit, the NIEHS, did assertively commit itself to embracing complexity analytically and environmental justice more than rhetorically. Had it been encouraged to do so, the NIEHS might with programmatic research have begun to lay an evidentiary basis for fundamental regulatory reform.

Mixture toxicity and related issues presented to regulators an infinitude of interactions — Silbergeld’s “googolplex” — whose sorting, both scientifically and legally, was impossible. Yet impossibility was not the problem. Impossibility was the message, one we can still read, and with ever sharper acuity. If toxicants cannot be accepted *within* the environment then toxicants must not be accepted *into* the environment. However facile it may seem initially, this deduction teaches — to industry, to agriculture, to a self-governing citizenry — an actionable lesson: minimize the exposome by, first, decelerating its expansion.

Achieving even this much would require a salient intrusion into policy-as-usual and a robust commitment to end science-by-statute. Presidential and congressional cooperation would be needed and could be slow in coming. Agency-level initiatives could be more promising.

We did confirm the importance of statutory constraint, but we also found statutory contrast and opportunity, most obviously in two bills enacted in 1996 and a third enacted in 2016. The Food Quality Protection Act required cumulative-risk assessment. The Safe Drinking Water Act Amendments required complex-mixture assessment with pesticides not excluded. And the Lautenberg Act required particular attention be paid to potentially exposed or susceptible subpopulations, opening a path to environmental-justice analytics and interventions. These three requirements, as supported by the persistent prodding of the National Research Council, could form a premise for action by a future, or by the current, EPA administrator. In parallel, the argument OSHA failed to make persuasively to the Supreme Court with respect to benzene in 1980 could be remade more broadly and with four decades of subsequent science to back it up; if so, then no-safe-dose toxicants could once again have their emissions driven toward zero, and low-dose exposures could come within the range of management.

Regulatory science agencies and research science agencies are different, but they are not obliged to be as different as they have become. The most immediate need, we found, is for toxicologists to work with — not apart from and not in opposition to — epidemiologists, physiologists, endocrinologists, pathologists, and field biologists, all laboring to protect the environment from us and us from the environment.

## Data Availability

Not applicable.

## Abbreviations

ACEs: Adverse childhood experiences
ATSDR: Agency for Toxic Substances and Disease Registry
CERCLA: Comprehensive Environmental Response, Compensation, and Liability Act of 1980
CDC: Centers for Disease Control and Prevention
CMG: Common mechanism group
CRA: Cumulative-risk assessment
DPR: California Department of Pesticide Regulation
EJ: Environmental justice
EPA: Environmental Protection Agency
FIFRA: Federal Insecticide, Fungicide, and Rodenticide Act of 1947
FQPA: Food Quality Protection Act of 1996
NCEH: National Center for Environmental Health
NEJAC: National Environmental Justice Advisory Council
NHANES: National Health and Nutritional Examination Survey
NIEHS: National Institute of Environmental Health Sciences
NRC: National Research Council
NTP: National Toxicology Program
OSHA: Occupational Safety and Health Administration
RFA: Request for Applications
SDWAA: Safe Drinking Water Act Amendments of 1996
SRP: Superfund Research Program
TSCA: Toxic Substances Control Act
